# Recurrent Lymphocytic Pleural Effusion as a Complication of Ventriculopleural Shunt Meningitis Caused by Cutibacterium Acnes

**DOI:** 10.7759/cureus.13270

**Published:** 2021-02-10

**Authors:** Abdelmohaymin A Abdalla, Ragda Ali, Mansi Oberoi, Paul Berger

**Affiliations:** 1 Internal Medicine, University of South Dakota Sanford School of Medicine, Sioux Falls, USA; 2 Internal Medicine, University of Khartoum, Khartoum, SDN

**Keywords:** cutibacterium acnes, pleural effusion, ventriculopleural

## Abstract

*Cutibacterium acnes* (*C. acnes*) is part of the normal flora and has been linked to many invasive and pleural infections. Though it is usually considered a contaminant bacterium, full antimicrobial therapy might result in the resolution of foreign body-related infections. In this report, we describe an infection that started as ventriculopleural shunt meningitis but was complicated by a recurrent lymphocytic pleural infection. Ultimately, there was a resolution of pleural effusions after treatment of *C. acnes*.

## Introduction

*Cutibacterium acnes (C. acnes)* is an anaerobic gram-negative bacteria that is part of the normal flora of the skin, gastrointestinal tract, and oral cavity [1]. The bacteria have been linked to many invasive infections, particularly orthopedic implants and other foreign bodies [2]. Very few case reports have been reported in the pleuropulmonary compartment [3-4]. In one study, ventriculoperitoneal (VPR) shunt infections with subsequent meningitis have been reported in 14.6% out of all shunt-related infections [5]. Here, we describe an interesting story of a 52-year-old patient who had successful resolution of recurrent pleural effusions related to ventriculopleural (VPL) shunt infection caused by penicillin-resistant *C. acnes*.

## Case presentation

A 52-year-old male presented to the emergency department with a two-week history of worsening headache and dizziness. Headaches were diffuse and poorly characterized, worse than prior headaches, and were associated with two episodes of non-bloody, non-bilious vomiting. He also felt the room spinning around him, and it was worse with leaning forward. He did not have fever, diplopia, hearing problems, or swallowing difficulty. He also had shortness of breath (SOB) with activity but denied orthopnea, cough, hemoptysis, chest pain, or lower extremity swelling. His home medications were aspirin and metoprolol.

Past medical history was significant for hypertension and traumatic brain injury (TBI) following a motor vehicle accident (MVA) 10 years ago. Surgical history was complex and started with VPR shunt placement after developing hydrocephalus following MVA. He also underwent revision of that VPR shunt three times over the last 10 years; the most recent one was six years ago. He also underwent VPL shunt placement six years ago after difficulties draining through the peritoneal one. He also had fenestrations of the fourth ventricle done 16 months ago for entrapped and progressively increasing fourth ventricular size.

On physical exam, he had no focal or new neurological deficit. His cardiovascular examination was normal, but he had diffuse dullness to percussion on the right side of the chest. Clinical examination was otherwise unremarkable.

**Figure 1:**
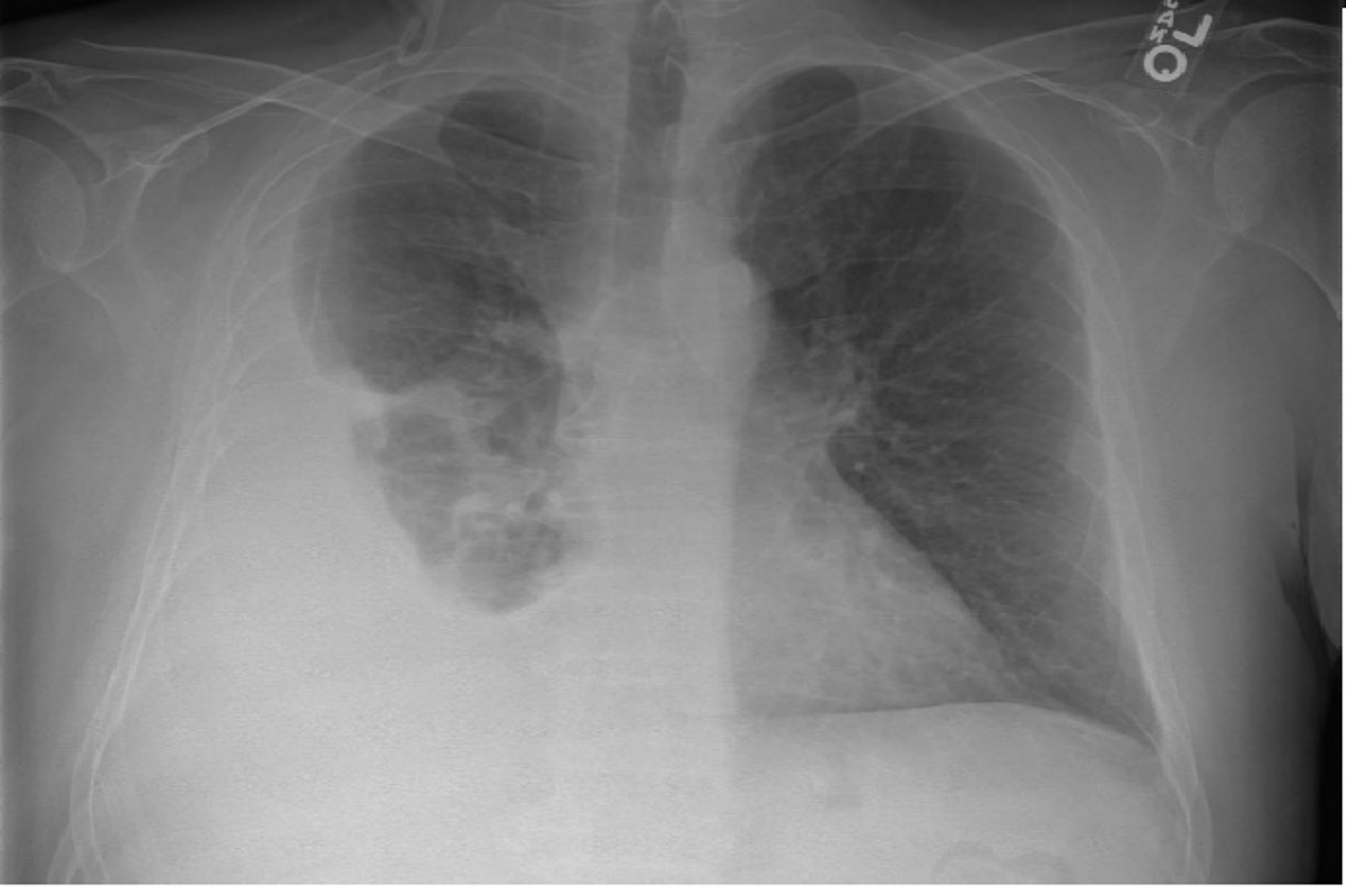
Chest X-ray showing a moderate-sized right-sided pleural effusion

**Figure 2:**
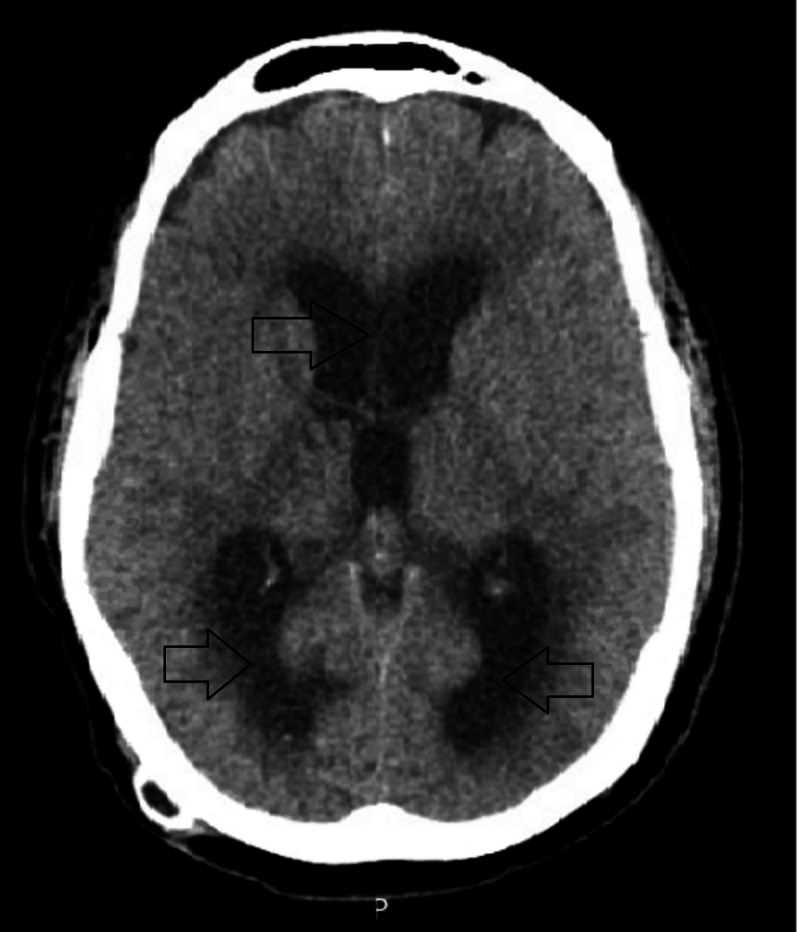
Computed tomography showing hydrocephalus that involves the lateral and third ventricles

The chest radiograph showed a right-sided moderate size pleural effusion (Figure 1). Complete blood count and erythrocyte sedimentation rate were normal. Thoracentesis revealed clear fluid that was exudative with a protein level of 3.4 g/dl (total serum protein of 5.9). The nucleated cell count was 1504, mainly lymphocytic (57%). Other lab values were within normal limits, and subsequent cultures and cytology were nonrevealing. The patient felt better after fluid removal, and his headaches were controlled with Tylenol. He was discharged home to follow closely with neurosurgery. Imaging of the brain with computed tomography (CT) a week later showed interval development of hydrocephalus involving the entire ventricular system (Figure 2).

The patient was readmitted later that week with increasing SOB and headache. Clinical examination and laboratory testing were almost identical to earlier admission. There was also a moderate-sized right-sided pleural effusion that was exudative and lymphocytic. Pleural fluid analyses were repeatedly unremarkable, including cytology and cultures (including mycobacterial and fungal cultures).

The following day, the neurosurgical team performed the conversion of VPL shunt into ventriculoatrial (VA) shunt. Intraoperative cerebrospinal fluid (CSF) samples were sent for laboratory analysis and culture. Cell count in the CSF was 22 (10 lymphocytes and 5 neutrophils). He was started on intravenous vancomycin for probable meningitis following repeated pleural effusion. Seven hours later, one of the CSF cultures grew gram-positive bacilli that were later identified as *C. acnes*. He then underwent removal of the VA shunt with confirmed repeat growth of *C. acnes* from intraoperative cultures. The patient had an external ventricular drain placed in the meantime.

Infectious disease consultation was sought and the decision was made to start ampicillin pending sensitivity testing, which later on showed penicillin-sensitive strains of *C. acnes*. Subsequently, he did have a re-collection of pleural fluid, necessitating chest tube/drain placement. For the third time, the pleural fluid was exudative and lymphocytic; yet, the cultures were negative. After seven days of ampicillin therapy, the chest tube was removed. His pleural effusion didn't recur following completion of antibiotic therapy and during the subsequent period of follow-up (two months).

## Discussion

Though it is common to identify *C. acnes* in the microbiology lab, these bacteria are often deemed contaminant. However, it has been increasingly recognized as the cause of solid organs infections and implants [6]. Pleural space infection has been reported by Lawrence et al. to result as a consequence of thoracoscopy [7]. Esteban et al. analyzed 118 samples of *C. acnes* in 1998, and zero of those were taken from pleural infections [6]. In a 2004 analysis, 198 samples of anaerobic pleural empyema were reviewed and not one was due to *C. acnes* [8].

In 2005, a European surveillance study announced that antimicrobial resistance has emerged among *C. acnes* isolates [9]. In 2013, a sampling of 98 patients showed that more than half of them were colonized with strains that are resistant to clindamycin and erythromycin [10]. In contrast, a study by Biswal et al. showed that only 10% of isolates were resistant to clindamycin [11]. In a recent prospective study, Zhu et al. demonstrated that many strains were sensitive to tetracyclines but higher resistant patterns were seen in patients who received topical or systemic antimicrobial therapy [12]. Most of the studies reviewed in the literature used a panel of isolates from the skin, commonly from patients diagnosed with (and/or undergoing therapy for) acne.

Despite the first identification of *C. acnes* in our patient, from the CSF culture, our initial thought was that it was a contaminant since the CSF cell count was not consistent with a bacterial infection. After the second isolation of *C. acnes* from the VA shunt, in addition to the lack of other findings to explain the concomitant infection/disease process, our final diagnosis was an infection of the pleural space with the same bacteria through VPL. This proposition is supported by the fact that the effusion did not recur (during the follow-up period of two months) after the completion of ampicillin therapy.

## Conclusions

A* C. acnes*-related VPL shunt infection might be difficult to isolate in pleural fluid cultures. If *C. acnes* is identified in the CSF in patients with a suspected VPL shunt infection, a full course of appropriate antimicrobial therapy might result in the resolution of pleural cavity infection and might be a successful treatment of recurrent associated pleural effusion.
